# Unusual giant multilocular mesothelial cyst of mediastinum

**DOI:** 10.1186/s40792-020-01011-8

**Published:** 2020-10-01

**Authors:** Takehiko Manabe, Soichi Oka, Kenji Ono

**Affiliations:** Thoracic Surgery, Kokura Memorial Hospital, Asano, Kokurakita-ku, Kitakyushu-shi, Fukuoka 802-8555 Japan

**Keywords:** Mesothelial cyst, Multilocular cyst, Giant mediastinal cyst, Mediastinal tumor

## Abstract

**Background:**

Intrathoracic mesothelial cysts are congenital lesions induced by the abnormal development of the pericardial coelom. There have been a few reports of giant mesothelial cyst of the superior mediastinum, but the preferred treatment remains a controversial topic. We herein report a rare case of successful removal of giant mesothelial cyst that was incidentally detected during a medical checkup.

**Case presentation:**

A 53-year-old man with a feeling of mild chest tightness was referred to our hospital for the evaluation of an abnormal shadow of the mediastinum on chest X-ray. Computed tomography showed a multilocular, homogenous, large cyst in the superior mediastinum measuring 18 cm in size without contrast enhancement and with spotty calcification, and magnetic resonance imaging showed a low intensity on T1-weighted images and high intensity on T2-weighted images. Therefore, a cystic thymoma, thymic cyst, lymphangioma, cystic teratoma or pericardial cyst was suspected as the preoperative diagnosis. Despite mild symptoms, the patient underwent total thymectomy under median sternotomy for an appropriate diagnosis and treatment. The pathological diagnosis was giant multilocular mesothelial cyst.

**Conclusions:**

Intrathoracic mesothelial cyst is a benign cyst and generally asymptomatic, but can sometimes induce critical chest clinical symptoms if untreated, depending on its size. In our case, complete surgical resection and a detailed pathological evaluation was effective for making the appropriate diagnosis and delivering treatment. In addition, an immunohistological evaluation is effective for diagnosing mesothelial cysts when it is difficult to distinguish the cyst from other cystic lesions.

## Background

The occurrence of mesothelial cyst of the mediastinum is rare, accounting for 3–6% of all mediastinum tumors. These cysts are usually asymptomatic and are of little clinical importance, being classified as congenital abnormalities [1]. They may sometimes enlarge without any symptoms and may induce dyspnea, pleural effusion, chest pain and orthopnea, some of which may conceal lung cancers, thymoma or other malignant tumors [2].

We herein report a rare case of a patient who underwent total thymectomy for a giant cyst of the upper mediastinum and was diagnosed with mesothelial cyst of the upper mediastinum by an immunohistological examination.

## Case presentation

**Fig. 1:**
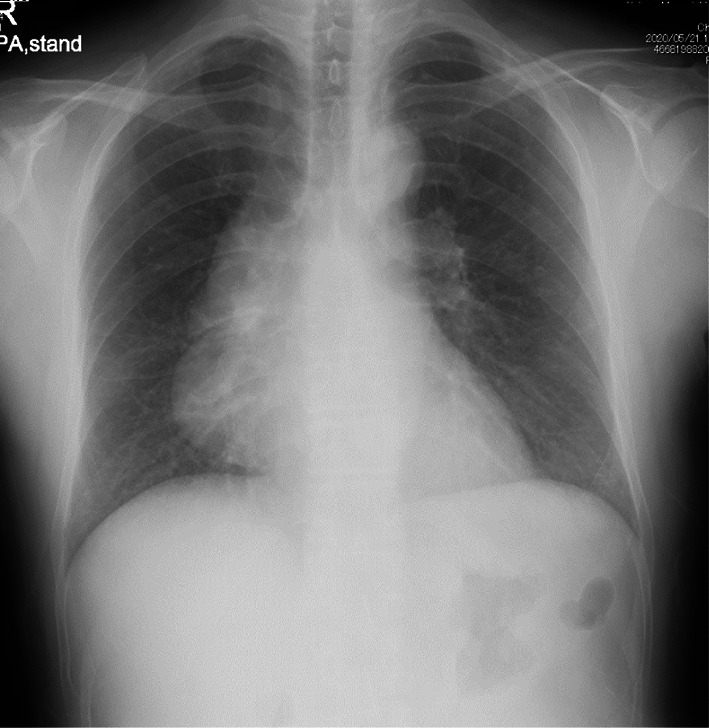
Preoperative chest X-ray showed an abnormal mass in the right pericardial region

The patient was a 53-year-old man who was referred to our hospital because of an abnormality at a medical checkup. He did not have a medical checkup for more than 5 years. He sometimes had a feeling of mild chest tightness. Chest X-ray showed an abnormal mass in the right pericardial region (Fig. 1). Physiological and laboratory examinations were normal. Tumor markers, including soluble IL-2 receptor, carcinoembryonic antigen (CEA) and alpha fetoprotein (AFP), were within the normal ranges. He had no remarkable medical history or history of trauma. He smoked one pack of cigarettes per day.

**Fig. 2:**
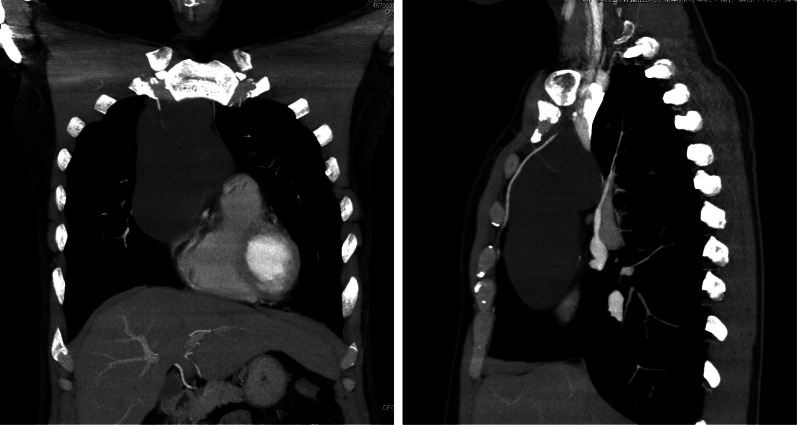
Enhanced CT revealed a lobulated homogenous giant mass in the superior-to-anterior mediastinum. The components of the cyst had increased CT attenuation of 10 HU, which is higher than that of water

**Fig. 3:**
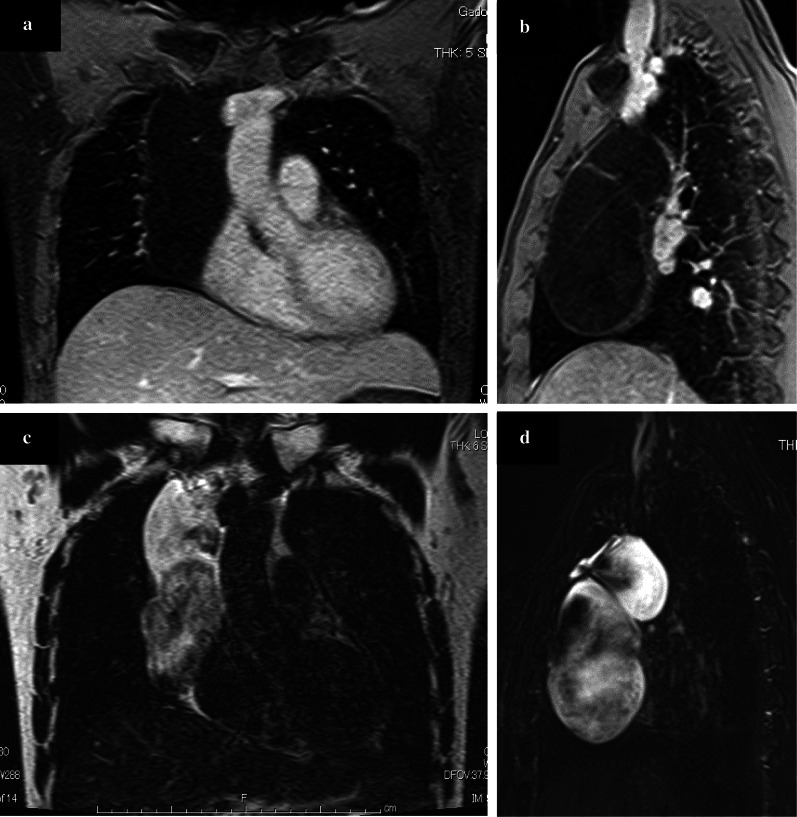
Chest MRI revealed the contents of a cyst with typical characteristic of fluid, i.e., **a**, **b** a low signal intensity on T1-weighted images and **c,** **d** high signal intensity on T2-weighted images

Preoperative computed tomography (CT) showed a large, superior-to-anterior mediastinal, fluid-filled and well-demarcated cyst. The cyst extended from the superior mediastinum caudally on the right atrium to 18 cm in size. The anterior–posterior component measured 5.6 cm. There were septations and spotty calcification, but no solid component in the cyst, which had a slightly greater CT attenuation than water (Fig. 2). The remainder of the findings were unremarkable. Chest magnetic resonance imaging (MRI) revealed that the lesion had a low signal intensity on T1-weighted images and high signal intensity on T2-weighted images and no findings of hemorrhaging or a solid component (Fig. 3). Radiologically, the provisional diagnosis was a cystic thymoma, thymic cyst, lymphangioma, cystic teratoma or pericardial cyst.

**Fig. 4:**
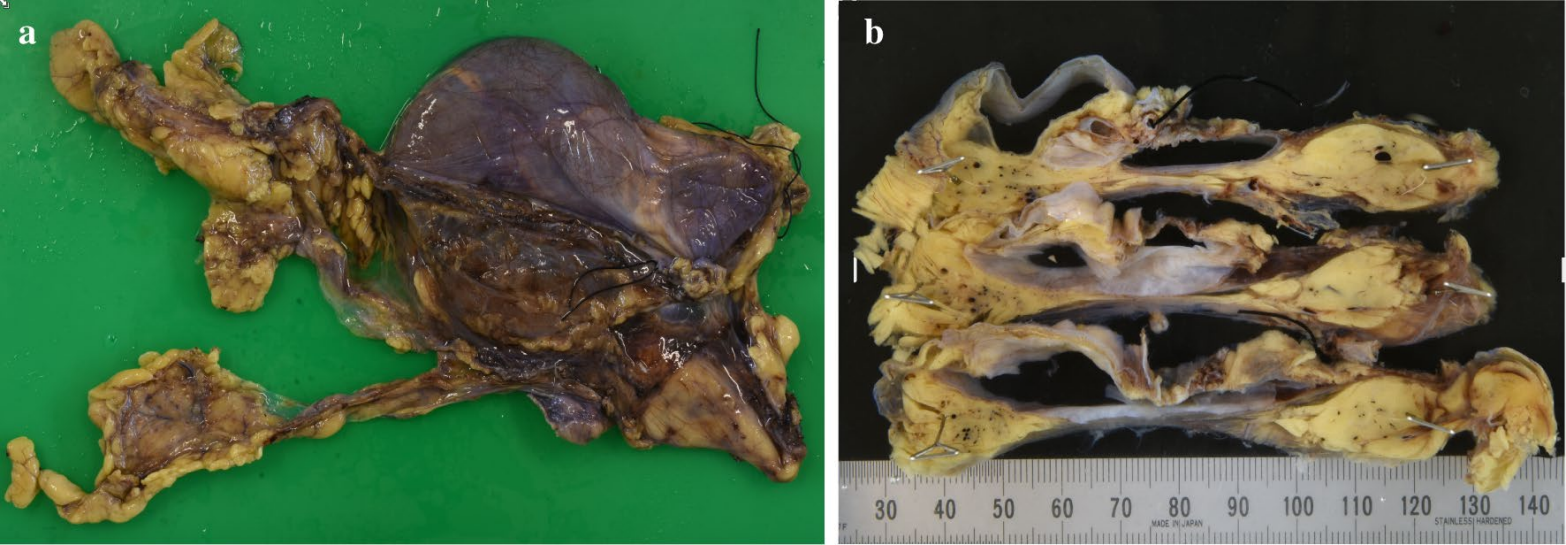
**a** Macroscopically, the lesion was 12 × 8.5 × 2 cm in diameter located at the upper pole of the thymus. **b** The cut surface of the cyst revealed a grayish-white, well-encapsulated and thin-walled cyst

**Fig. 5:**
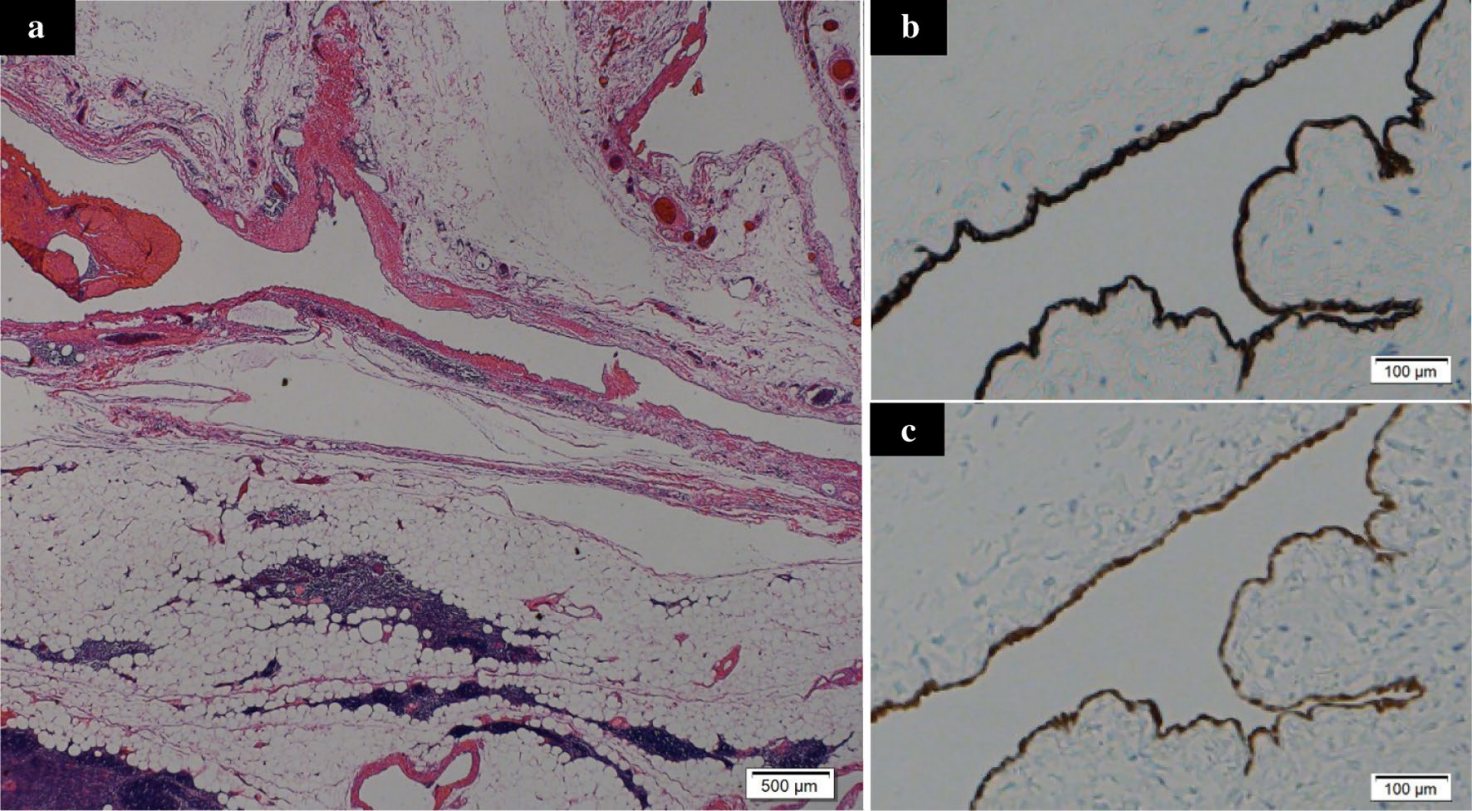
Microscopically, the cyst wall was lined by cubic cells underlaid by loose connective tissue. The remnants of the thymus were not adjacent to the cyst wall (**a**). Immunohistologically, the cyst consisted of a thin wall with a single layer of mesothelial cells positive for cytokeratin 7 (**b**) and calretinin (**c**)

Despite the mild state of his symptoms, we decided to surgically remove the cyst because of the possibility of the coexistence of varied malignancies or the induction of critical manifestations if it ruptured, including hemorrhaging or compression of the surrounding structures. Total thymectomy under mediastinal sternotomy was performed with the successful removal of the intact cyst. During the surgery, a well-encapsulated and thin-walled cyst filled with serous fluid was observed in the superior-to-anterior mediastinum that adhered to the internal thoracic artery but did not invade it. We did not need to divide any thoracic major vessels because the cyst was separated from them. The tumor was 15 × 8.5 × 2 cm in size with grayish-white discoloration (Fig. 4a). The cut surface revealed a multilocular cyst in the right upper pole of thymus (Fig. 4b). In addition, a microscopic examination of the tumor showed that the cyst was lined by cuboid cells and underlying loose connective tissue with remnants of thymic tissue (Fig. 5a). Immunohistologically, the epithelial lining of the cyst was diffusely positive for calretinin, and there was cytoplasmic positivity for cytokeratin 7 (Fig. 5b) and calretinin (Fig. 5c), confirming mesothelial differentiation. Similarly, the septum was made of cuboidal cells with the same immunohistological findings as the cyst wall. No malignancy was identified.

Based on these findings, the pathological diagnosis of intrathoracic mesothelial cyst of the mediastinum was made. The patient was discharged without any post-operative complications 7 days after the operation, and was followed up for 2 months post-operatively without any recurrence.

## Comment

Mesothelial cysts are benign entities that are classified as congenital tumors and usually diagnosed in the fourth to fifth decade of life. They are generally unilocular, small and contain clear fluid within a thin wall. As their classic anatomical location is in the cardio-phrenic angle, they are also referred to as pleuro-pericardial cysts. The incidence of unusual locations, such as the chest wall, mediastinum and diaphragm, is between 8 and 11% [3]. Recently, with the increasing rate of health checkups and advances in imaging diagnostic technology, such as the advent of thin-slice CT, the diagnosis and anatomical evaluation of these lesions has improved, and reports of unusual locations have shown an increasing trend [4].

The atypical locations of mesothelial cysts have embryological causes, as these cysts are congenital lesions due to an anomaly in the development of the pericardial coelom. During development, incomplete fusion or secondary migration of isolated elements, such as a lacuna, can occur at the level of the parietal pleura, mediastinal pleura or septum transversum, which explains the unusual locations of mesothelial cysts [5]. On occasion, these cysts in the mediastinum may extend outside the confines of the mediastinal proper, allowing the lesion to compress adjacent structures, as shown in our case [1].

In the diagnosis of mesothelial cysts, we cannot easily distinguish preoperatively whether or not a cystic lesion is another type of mediastinal cyst. In our case, since the cyst was located in the right upper pole of the thymus and uncommonly multilocular, it was difficult to distinguish it as another tumor, such as a thymic cyst, which is derived from thymic tissue. Although mesothelial cysts are usually unilocular [3], Cangemi et al. [5] reported that 23 of 24 pericardial cysts were unilocular, and 1 was multilocular. For this reason, mesothelial cyst should be included in the differential diagnosis, regardless of locularity. Furthermore, the immunohistological stains has an important role in the diagnosis of mesothelial cyst. In recent reports of mesothelial cyst, the diagnosis was mostly established based on immunohistological findings; the epithelium of the cyst wall was lined by mesothelial cells [1]. Our diagnosis of mesothelial cyst was based on the fact that the epithelium of the cyst was lined by cubic cells positive for calretinin and cytokeratin 7 on staining, which indicates mesothelial differentiation and the pleural origin, and the cyst was separated from the remnants of the thymic tissue. Therefore, surgical resection with an immunohistological evaluation can help us precisely analyze the characteristics of a tumor and lead to the elucidation of its origin and pathology.

Although consensus- and evidence-based guidelines on the management of giant mesothelial cysts are currently lacking, therapeutic treatment, such as puncture or extirpation of the cyst, is occasionally necessary. We recommend removing a cyst surgically when encountering a giant benign mediastinal cyst for which the diagnosis is unclear, for two reasons. First, there is the possibility that the benign cyst may mask other pathological changes of the thymus or other malignant tumors. In some reports, benign mediastinal cysts, such as thymic cysts, which have similar radiological and pathological findings to mesothelial cysts, have been associated with various malignancies, including thymic carcinoma, thymoma and lung carcinoma [6]. Surgical resection is needed in order to obtain a definitive diagnosis and avoid missing other life-threatening tumors, although intrathoracic mesothelial cyst is less likely to be associated with malignant tumors. Second, the complete removal of a cyst should be considered when there is a possibility of serious complications occurring. From a clinical perspective, although more than 50% of mesothelial cysts are asymptomatic, critical clinical symptoms can be induced by the compression of surrounding structures (like the heart, great vessels and lungs), infection, hemorrhaging and rupture, which sometimes provoke arrhythmias, cardiac standstill and respiratory failure [3]. Furthermore, in some cases, the percutaneous drainage of the cyst fluid has been shown to be effective for alleviating symptoms when patients were not suitable for surgery [1, 7]. However, surgical excision is the ideal treatment of these lesions to eliminate the possibility of recurrence or other collateral damage, such as cyst infection.

## Conclusions

We herein report a rare case of an unusual large multilocular mesothelial cyst of the superior mediastinum that was successfully extirpated. For the accurate diagnosis and treatment, asymptomatic large benign cysts carrying a risk of exerting compressive effects in the near future or concealing malignancies should be surgically removed. Furthermore, immunohistological examinations will support the correct diagnosis of mesothelial cysts.

## Data Availability

Not applicable.

## Abbreviations

CT: Computed tomography
soluble IL-2 receptor: Soluble interleukin-2 receptor
CEA: Carcinoembryonic antigen
AFP: Alpha fetoprotein
MRI: Magnetic resonance imaging
